# RETRACTED: Alluri et al. Repurposing Diltiazem for Its Neuroprotective Anti-Dementia Role Against Intra-Cerebroventricular Streptozotocin-Induced Sporadic Alzheimer’s Disease-Type Rat Model. *Life* 2023, *13*, 1688

**DOI:** 10.3390/life16071175

**Published:** 2026-07-16

**Authors:** Ramesh Alluri, Eswar Kumar Kilari, Praveen Kumar Pasala, Spandana Rajendra Kopalli, Sushruta Koppula

**Affiliations:** 1 Cognitive Science Research Initiative Lab., Department of Pharmacology, Vishnu Institute of Pharmaceutical Education and Research, Medak Dist., Narsapur 502313, India; 2 Department of Pharmacology, University College of Pharmaceutical Sciences, Andhra University, Visakhapatnam 530003, India; ekilari@gmail.com; 3 Department of Pharmacology, Raghavendra Institute of Pharmaceutical Education and Research, Jawaharlal Nehru Technological University Anantapur—JNTUA, Anantapur 515721, India; praveenpharmaco@gmail.com; 4 Department of Integrated Bioscience and Biotechnology, Sejong University, Gwangjin-gu, Seoul 05006, Republic of Korea; spandanak@sejong.ac.kr; 5 College of Biomedical and Health Science, Konkuk University, Chungju-si 380-701, Republic of Korea

The journal retracts the article titled “Repurposing Diltiazem for Its Neuroprotective Anti-Dementia Role against Intra-Cerebroventricular Streptozotocin-Induced Sporadic Alzheimer’s Disease-Type Rat Model” [1], cited above.

Following publication, concerns were brought to the attention of the Editorial Office regarding the integrity of figures presented in this article [1].

In accordance with the journal’s standard procedures, the Editorial Office and Editorial Board conducted an investigation, which identified indications of inappropriate image editing and duplication in Figure 7. Although the authors cooperated with the investigation, the explanations and supporting materials provided were not deemed sufficient by the Editorial Board to resolve the identified concerns. As a result, the Editorial Board has lost confidence in the reliability of the findings and has decided to retract this publication [1], in line with MDPI’s retraction policy.

This retraction was approved by the Editor-in Chief of the journal *Life*.

The authors have been informed of this retraction and have indicated their agreement with this decision.
